# Arsenic Trioxide Overcomes Rapamycin-Induced Feedback Activation of AKT and ERK Signaling to Enhance the Anti-Tumor Effects in Breast Cancer

**DOI:** 10.1371/journal.pone.0085995

**Published:** 2013-12-31

**Authors:** Cynthia Guilbert, Matthew G. Annis, Zhifeng Dong, Peter M. Siegel, Wilson H. Miller, Koren K. Mann

**Affiliations:** 1 Lady Davis Institute for Medical Research, McGill University, Montreal, Canada; 2 Rosalind and Morris Goodman Cancer Research Centre, McGill University, Montreal, Canada; 3 Department of Oncology, McGill University, Montreal, Canada; Dartmouth, United States of America

## Abstract

Inhibitors of the mammalian target of rapamycin (mTORi) have clinical activity; however, the benefits of mTOR inhibition by rapamycin and rapamycin-derivatives (rapalogs) may be limited by a feedback mechanism that results in AKT activation. Increased AKT activity resulting from mTOR inhibition can be a result of increased signaling via the mTOR complex, TORC2. Previously, we published that arsenic trioxide (ATO) inhibits AKT activity and in some cases, decreases AKT protein expression. Therefore, we propose that combining ATO and rapamycin may circumvent the AKT feedback loop and increase the anti-tumor effects. Using a panel of breast cancer cell lines, we find that ATO, at clinically-achievable doses, can enhance the inhibitory activity of the mTORi temsirolimus. In all cell lines, temsirolimus treatment resulted in AKT activation, which was decreased by concomitant ATO treatment only in those cell lines where ATO enhanced growth inhibition. Treatment with rapalog also results in activated ERK signaling, which is decreased with ATO co-treatment in all cell lines tested. We next tested the toxicity and efficacy of rapamycin plus ATO combination therapy in a MDA-MB-468 breast cancer xenograft model. The drug combination was well-tolerated, and rapamycin did not increase ATO-induced liver enzyme levels. In addition, combination of these drugs was significantly more effective at inhibiting tumor growth compared to individual drug treatments, which corresponded with diminished phospho-Akt and phospho-ERK levels when compared with rapamycin-treated tumors. Therefore, we propose that combining ATO and mTORi may overcome the feedback loop by decreasing activation of the MAPK and AKT signaling pathways.

## Introduction

The PI3K/AKT/mTOR pathway is constitutively-activated in many tumor types leading to enhanced tumor survival. Thus, mTOR complexes appear to be attractive targets for novel therapeutics. Several novel rapamycin derivatives, collectively known as rapalogs, have shown exciting clinical activity in renal cell carcinoma [1], breast cancer [2], and hematologic malignancies [3]. Sensitivity to mTOR inhibitors requires an active PI3K/AKT/mTOR pathway. As part of this pathway, AKT phosphorylation disrupts the Tuberous Sclerosis Complex (TSC), which can no longer inhibit RHEB-GTPase activity, resulting in mTOR activation. AKT can also phosphorylate PRAS40 (proline-rich Akt substrate of 40 kDa) causing it to dissociate from mTOR and relieve its inhibitory activity [4]. mTOR exists in two complexes: mTORC1 and mTORC2[5]. Both complexes contain mTOR and GβL, but mTORC1 contains RAPTOR, while mTORC2 contains RICTOR. Rapalogs bind and inhibit the activation of the mTOR complex, mTORC1, and its subsequent activation of eIF4e, p70S6 kinase, and other genes involved in translational regulation, protein synthesis and metabolism. However, the potential benefits of rapalogs are limited by a feedback mechanism that results in AKT activation. While rapalogs can block important growth promoting events downstream from mTORC1, an increased activation of AKT may inhibit apoptotic signals [6,7]. Although rapalogs inhibit cell cycle progression mediated by mTORC1, feedback activation of AKT can inhibit apoptotic signaling of the MAPK cascade[8–10], as well as initiate other AKT-dependent pro-survival pathways. 

The exact nature of this feedback mechanism is unknown, although several models have been suggested. It has been postulated that the increased AKT activity in response to rapalog treatment is a result of increased IGF signaling via IRS-1 in breast cancer or IRS-2 in leukemia [6,11]. Alternatively, AKT activation may occur via the second mTOR complex, mTORC2. mTORC2 is less sensitive to inhibition by rapamycin [12], but with prolonged treatment, the mTORC2 complex may be disrupted [13]. In any case, a rapid increase in phospho-AKT levels has been seen in malignant cell lines treated with rapalogs [6]. Translational clinical trials using multiple serial biopsies confirm an activated AKT response in the malignant cells of patients treated with rapalogs [7].

Finally, mTOR serves as an integration point of the PI3K signalling pathway and the MAPK/ERK pathway. The MAPK/ERK pathway phosphorylates the TSC proteins reducing their ability to inhibit mTORC1 [14,15]. In turn, rapalog-mediated inhibition of mTOR increases ERK activation both *in vitro* and in tumor biopsies from patients treated as part of a clinical trial [16]. Thus, activation of ERK may be a mechanism of resistance to rapalogs. Indeed, combination therapy with MEK inhibitors, which block activation of ERK, enhances the anti-tumor effects of mTORi [17,18]. Thus, an important goal for future clinical trials is to find a drug combination that can inhibit AKT concomitant with decreased mTOR signaling, while also inhibiting the activation of the MAPK/ERK pathway. 

Arsenic trioxide (ATO) is used as part of standard therapy for acute promyleocytic leukemia (APL). In APL, ATO acts in part through differentiation of the accumulated promyelocytic blasts. In APL, as well as other malignant cell types, ATO induces apoptosis through a mechanism that requires activation of the SEK/JNK signalling cascade. We and others have shown that ATO treatment leads to decreased AKT activity and protein expression [19–21]. Over-expression of constitutively active AKT constructs inhibits ATO-induced apoptosis [19]. We hypothesize that ATO may block survival signals that are engaged in response to rapalog treatment, leading to better anti-tumor effects with the drug combination. 

We tested whether ATO treatment could enhance the efficacy of mTORi *in vitro* and *in vivo*. Treatment of breast cancer cells with a combination of mTORi and ATO revealed that ATO could enhance the efficacy of mTORi in some cell lines. Enhanced efficacy *in vitro* corresponded with the ability to decrease mTORi-induced phosphorylation of AKT. Furthermore, we tested the combination in an MDA-MD-468 xenograft model. The combination of mTORi and ATO resulted in significantly enhanced anti-tumor activity, without a significant increase in ATO-induced hepatotoxicity. Furthermore, the increased anti-tumor activity corresponded with decreased AKT and ERK phosphorylation.

## Materials and Methods

### Reagents

Rapamycin (RAP) was purchased from LC Laboratories and resuspended in 100% ethanol at 50mg/mL. PEG 400 (20% solution in 0.1M calcium acetate and 100mM HEPES, pH 7) and TWEEN 80 were purchased from Sigma. As_2_O_3_ (ATO; Sigma) was resuspended at 0.15M in 0.4N NaOH, and diluted in phosphate-buffered saline (PBS) so that the final vehicle concentration was 5.3 X 10^-6^N NaOH A 25 mg/mL temsirolimus (TEM) solution was obtained from Wyeth and diluted to 10 mg/mL using ethanol; further dilutions were done in PBS so that the final concentration of vehicle was 2.5 x 10^-5^ % EtOH. 

### Cell culture

MDA-MB-468, MCF-7, SkBr3 and T47D breast cancer cell lines were obtained from ATCC and cultured in DMEM supplemented with 10% fetal bovine serum and penicillin/streptomycin. 

### Propidium iodide staining

Propidium iodide (PI) staining was performed as previously described [22]. Cells were seeded either at 40,000 cells per well for MDA-MB-468, or 35,000 cells per well for MCF-7, SkBr3 and T47D in 12 well plates and treated for 48h with vehicle control, 1µM ATO and/or 5ng/mL TEM. Fluorescence was detected using a FACS Calibur flow cytometer and analysis carried out using FCS Express software (BD Biosciences). Apoptotic cells were defined as events with PI fluorescence weaker than the G_0_-G_1_ cell cycle peak (the subG_0_ peak) when assessed on logarithmic scale. Cell cycle was assessed on a linear scale. 

### Annexin-V PE staining

SkBr3 cells were seeded at 35,000 cells per well in 12 well plates, left to adhere overnight and treated for 48h with vehicle control, 2µM ATO and/or 5ng/mL TEM. Annexin-V PE staining (BD Biosciences) was performed according to manufacturer's instructions. Fluorescence was detected using a FACS Calibur flow cytometer and analysis carried out using FCS Express software (BD Biosciences). Apoptotic cells were defined as those bound by Annexin V, but not labeled by 7-AAD.

### Sulforhodamine B (SRB) Colorimetric Assay

Inhibition of cell growth was measured using SRB colorimetric assay. Cells growing in log phase were seeded at 5,000 cells per well in a 24 well plate on day 0 and left to adhere overnight. The next day, cells were treated with media, 2 µM ATO and/or 5 ng/mL TEM for 48h. Then, cells were fixed by gently adding trichloroacetic acid to the cell culture media at a final concentration of 10% and incubated for 30 minutes at 4°C. Following fixation, cells were washed 4 times with distilled water and air-dried overnight at room temperature. The cells were stained with 400 µL of 0.4% SRB (Sigma) in 1% acetic acid for 30 minutes with gentle shaking. Following staining, cells were washed 4 times with 1% acetic acid and air-dried overnight. Then, cells were dissolved in 10 mM unbuffered Tris base (pH 10.5) and incubated for 10 minutes with shaking. 100 µL of dissolved sample was transferred to a 96 well plate and absorbance was measured at 570 nm. Experiments were done in triplicate. 

### Immunoblotting

One million breast cancer cells were seeded per 100mm dish on day 0 and left to adhere overnight. The next day, cells were treated with either vehicle control, 1-2 µM ATO, 0.5-5 ng/mL TEM or combinations for 24h. Cells were washed with cold PBS and lysed in radioimmunoprecipitation assay buffer and immunoblotting was performed as previously described [19]. More specifically, 50µg protein was separated on 7.5% SDS-PAGE gels and transferred to nitrocellulose. Membranes were incubated overnight with primary antibodies: phospho-AKT (S473) (1:500, Cell Signaling), phospho-AKT (T308) (1:750, Cell Signaling), AKT (1:750 Cell Signaling), phospho-p44/42 MAPK (T202/Y204) (1:1000, Cell Signaling), ERK2 (1:2000, SantaCruz), phospho-S6 (1:1500, Cell Signaling), S6 (1:1000, Cell Signaling), and β–actin (1:5000, Sigma). From the xenograft model, protein was extracted from flash frozen tumors, and 50 µg of protein was resolved on a 10% SDS-PAGE gel and immunoblotted as described above. Densitometry was performed using the ImageJ software (National Institute of Health). 

### Ethics statement

All animal experiments were conducted according to the protocol approved by Mcgill University Animal Care Committee.

### MDA-MB-468 xenograft mouse model

Five million MDA-MB-468 cells were injected into the fourth mammary fat pad of anaesthetized SCID/beige mice in 50:50 PBS: Matrigel (Becton Dickinson). Mice were treated with analgesics prior to and for three days following tumor implantation and housed for the duration of the study in specific pathogen-free conditions in individualized caging. For molecular analysis, mice were randomized into treatment groups when tumors reached 61-155 mm^3^. For tumor regression analyses, mice were randomized to treatment group when tumors reached 100-200 mm^3^. Four treatment groups were included: vehicle control (20% PEG 400 and 20% TWEEN 80), 7.5mg/kg ATO, 7.5mg/kg rapamycin, or ATO plus rapamycin. Rapamycin was first solubilized in ethanol at 50 mg/ml and further dissolved in vehicle (20% PEG 400 and 20% TWEEN 80). Mice (n =8-9 per treatment) were treated by IP injection every other day. No toxicities were observed in the vehicle-treated animals. Tumors were measured twice a week and weight of animals was recorded once a week. Mice were euthanized by CO_2_ asphyxiation at the experimental endpoint (day 62) or when mice displayed any of the following symptoms or signs of distress: weight loss exceeding 20% of baseline bodyweight; body condition score (BCS) less than 2; hunched posture, lethargy or lack of grooming; a tumor mass that is ulcerated, necrotic or impairing normal function (e.g. eating or drinking) or exceeding acceptable size endpoints (2000 mm^3^ or 10% of the baseline bodyweight). Animals were monitored every other day.

Tumors were excised and split in two. Half of the tumor was fixed in 4% paraformaldehyde and embedded in paraffin for immunohistochemistry the other half flash frozen for protein extraction. Livers were also flash frozen for further analysis.

### Immunohistochemistry

Paraffin sections from 4-5 tumors from individual mice were subjected to antigen retrieval in 10 mM citrate buffer (pH 6.0) for 10 min at sub-boiling temperatures. Slides were incubated overnight at 4°C with: anti-Ki67 [1:100, Abcam (AB15580)] and phospho-AKT (S473) [1:50, Cell Signaling (9271)]. Following incubation with the primary antibody, a secondary biotin-conjugated antibody was applied for 30 min. After washing with distilled water, slides were developed with diaminobenzidine (Dako) as the chromogen. All slides were counterstained with Harris modified hematoxylin and scanned using a Scanscope XT digital slide scanner (Aperio, Vista, CA). Scanned images were analyzed using algorithms provided with the Imagescope software (Aperio, Vista, CA). Between 4,000 to 250,000 nuclei per tumor sample were scored with only the viable area of the tumor and thus, the larger the viable area, the more tumor was analyzed. Data are represented as mean +/- standard error. Viable tumor area was calculated as the area of Ki67 positive tumor tissue over total tumor area.

### Statistics

All *in vitro* experiments were repeated at least three times and statistical analysis were done with Prism version 5.0 (GraphPad) using a one-way ANOVA with post-hoc analyses. Statistical analysis of differences in final tumor volume was calculated using a two-tailed T-test. To calculate the specific growth rate (SGR) of tumor, the following formula was used: SGR = ln(V2/V1)/(t2-t1) where V2 is the final tumor volume and V1 is the initial tumor volume and t is time [23]. 

## Results

**Figure 1:**
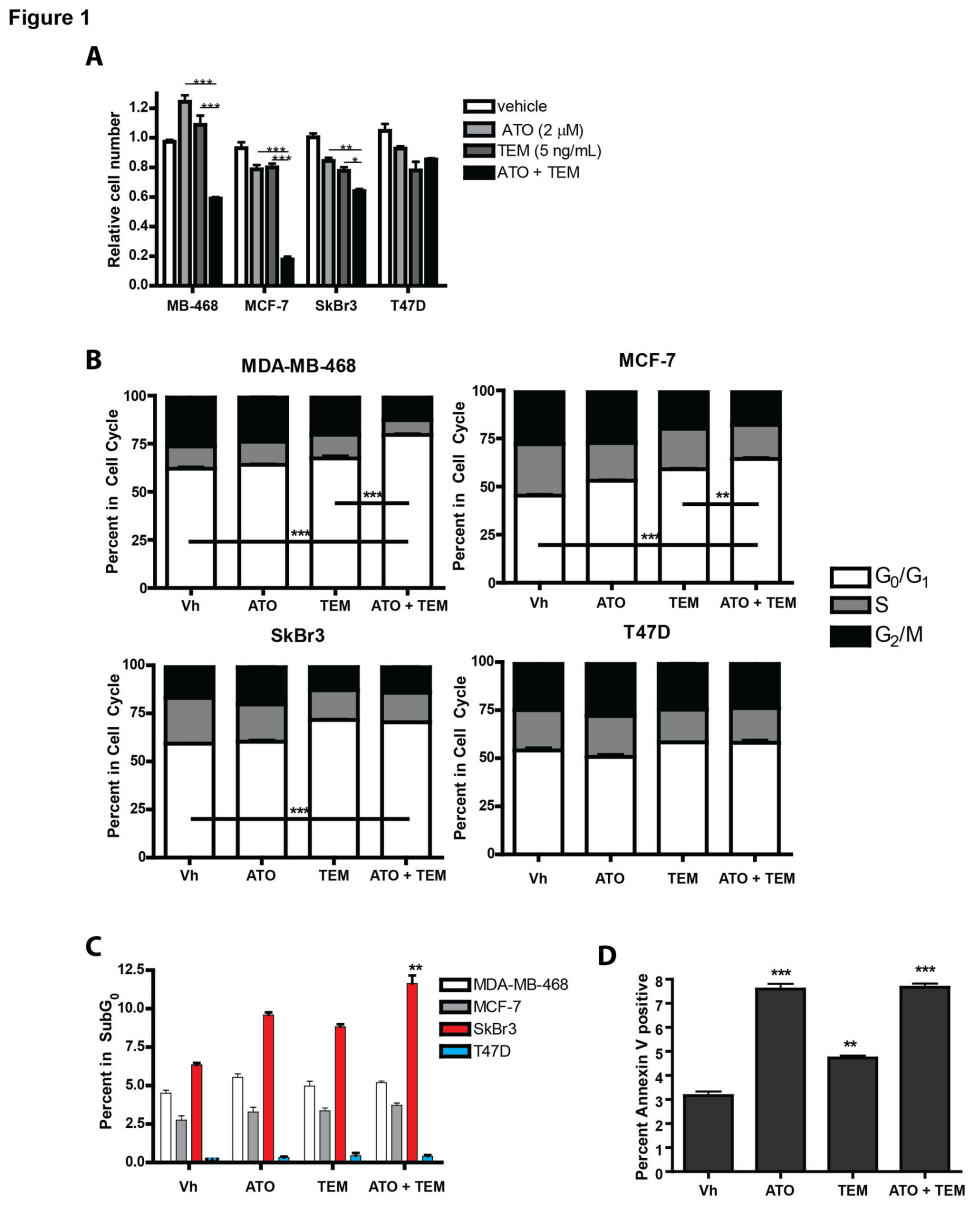
Arsenic enhances temsirolimus-induced growth inhibition of selective breast cancer cell lines. (A) MDA-MB-468, MCF-7, SkBr3, and T47D cell lines were exposed to vehicle control (Vh; 5.3 x 10^-6^ N NaOH and 2.5 x 10^-5^ % EtOH in PBS), 2 µM arsenic trioxide (ATO), 5 ng/ml temsirolimus, or the combination for 48 hours and relative cell number assessed by SRB assay. (B&C) Cell lines were exposed to vehicle control (Vh), 1 µM ATO, 5 ng/ml temsirolimus, or the combination for 48 hours and stained with propidium iodide and assessed by flow cytometry for cell cycle analyses (B) or percentage of cells with fragmented DNA (C). Annexin V staining was performed in SkBr3 cells and the apoptotic cells were determined as those staining positive for annexin V, but negative fot 7AAD. Statistical significance is denoted as follows: * = p<0.05, ** = p<0.01, and *** = p<0.001. Experiments were performed at least three times with technical triplicates.

Rapalog-mediated anti-tumor effects may be limited by activation of feedback mechanisms resulting in survival signals, including engagement of both the AKT and ERK signaling pathways. Our previous *in vitro* studies showed that ATO treatment of several cell types including breast cancer cell lines resulted in decreased AKT signaling. We hypothesized that concomitant treatment of a rapalog with ATO would increase the cytotoxicity in cell lines, and this would correlate with decreased survival signals. We treated a panel of 4 breast cancer cell lines, MDA-MB-468, MCF-7, SkBr3, and T47D, with the rapalog temsirolimus, ATO, or the combination and assessed cell growth and cell death. These cell lines represent the heterogeneity of the disease including the luminal A cell lines MCF-7 and T47D, both which are ER positive, the PTEN-defective, basal cell line MDA-MD-468, and the Her2-positive SkBR3 cell line [24]. Cell growth was assessed after 48 hours of treatment by SRB assay, a quantitative measure of protein content reflective of cell number. In three cell lines, MDA-MB-468, MCF-7, and SkBr3, the combination of temsirolimus and ATO inhibited growth significantly more than either drug alone (Figure 1A). In contrast, the combination failed to enhance growth inhibition in the T47D cell line to a greater degree than either drug alone. Due to the potential for rapalogs to reduce protein synthesis, SRB assay was confirmed by TiterGlo assay that measures ATP levels. We next determined whether this decrease in cell growth was associated with cell cycle changes or apoptosis. Temsirolimus plus ATO resulted in a significant increase in the percentage of cells in the G0/G1 phase of cell cycle in both the MDA-MB-468 and MCF-7 cell lines, but not the T47D cell line (Figure 1B). In SkBr3 cells, the combination treatment did not significantly change the temsirolimus-induced increase in G0/G1, but rather the enhanced growth inhibition was due to an increase in the number of cells in the subG0 population representing cells with fragmented DNA (Figure 1C). Annexin V staining confirmed apoptosis was induced by ATO, TEM, and the combination in SkBr3 cells (Figure 1D). These results indicate that growth inhibition by temsirolimus plus ATO correlates with increased cell cycle arrest (MDA-MD-468 and MCF-7) or increased apoptosis (SkBr3).

**Figure 2:**
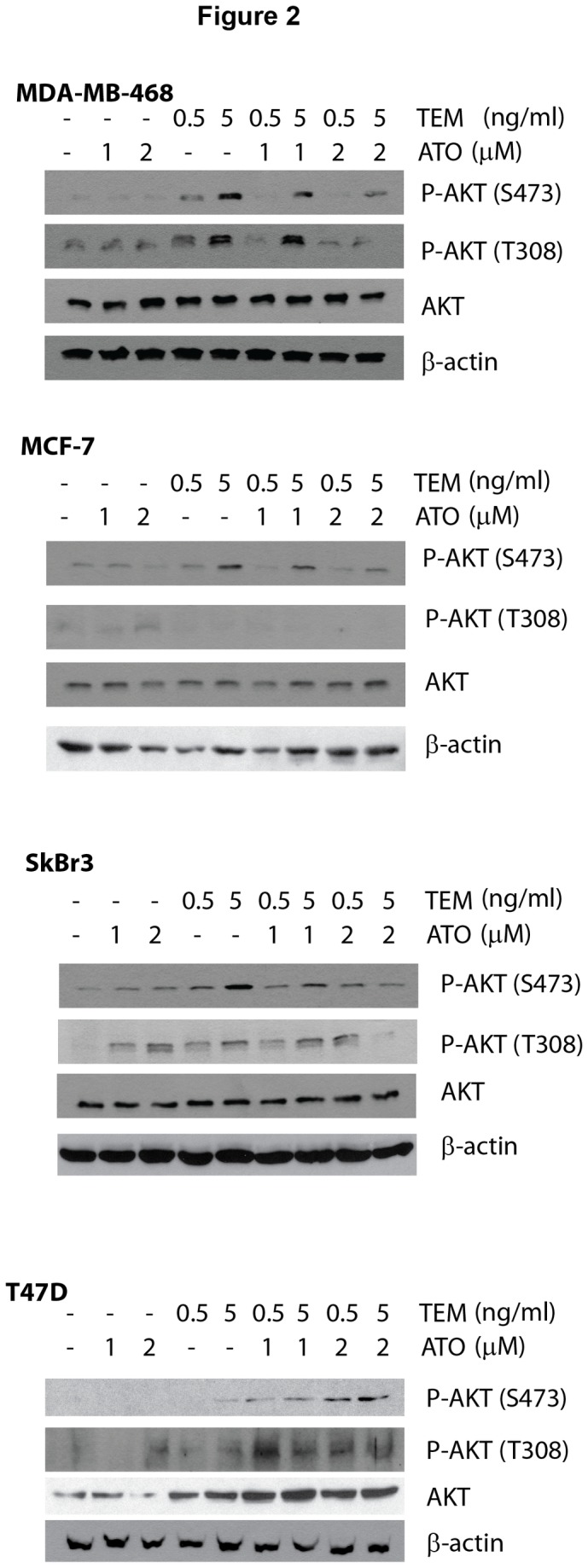
Arsenic growth inhibition correlates with decreased temsirolimus-induced phospho-AKT. MDA-MB-468, MCF-7, SkBr3, and T47D cell lines were exposed to vehicle control (Vh; 5.3 x 10^-6^ N NaOH and 2.5 x 10^-5^ % EtOH in PBS), 1-2 µM ATO, 0.5-5 ng/ml temsirolimus and the combinations for 24 hours. Whole cell extracts were used in immunoblotting experiments for phospho-S473-AKT, phospho-T308-AKT, AKT, and β-actin. Immunoblots shown are representative of experiments performed at least 3 times.

**Figure 3:**
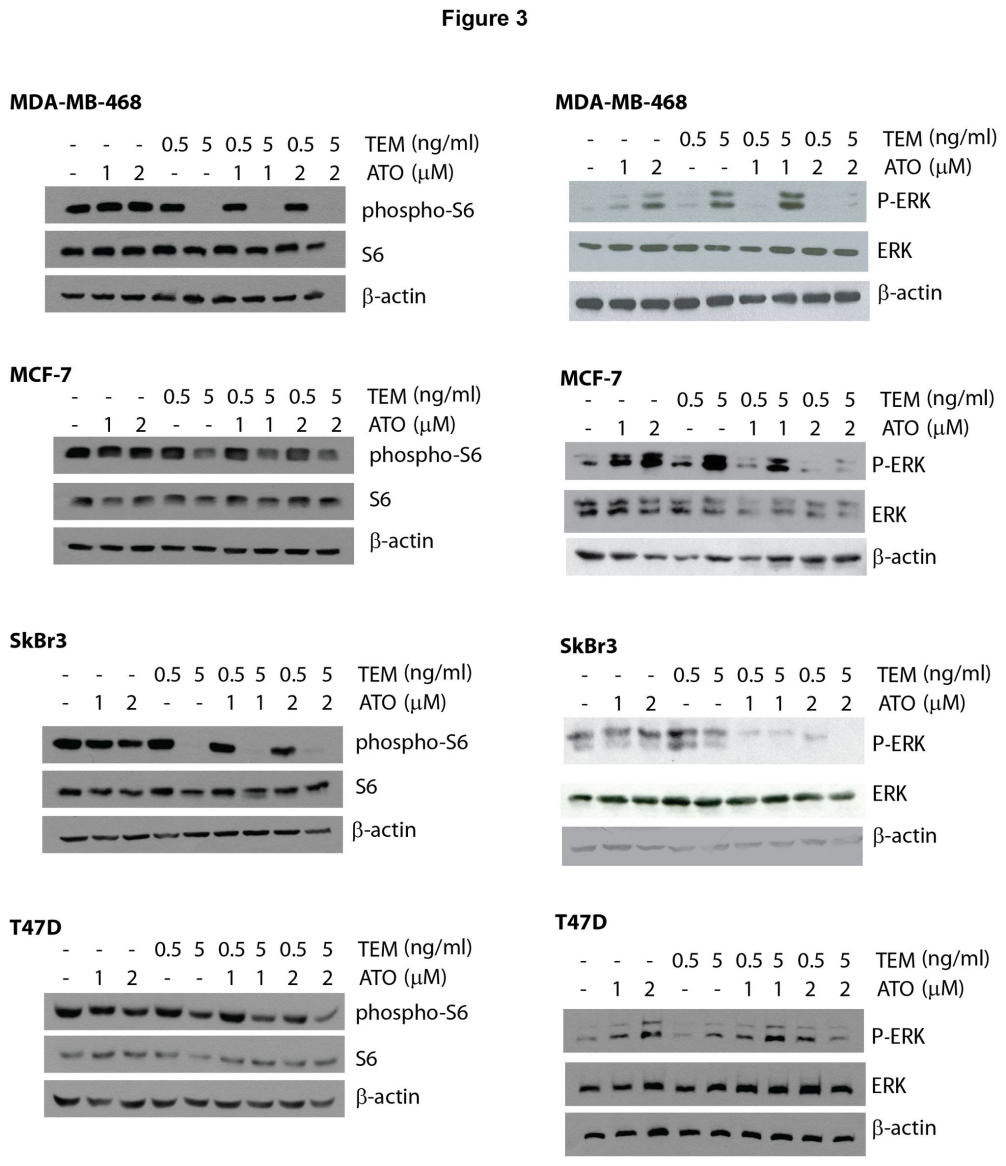
Arsenic and temsirolimus combination treatment results in decreased phospho-S6 and ERK activation. MDA-MB-468, MCF-7, SkBr3, and T47D cell lines were exposed to vehicle control (Vh; 5.3 x 10^-6^ N NaOH and 2.5 x 10^-5^ % EtOH in PBS), 1-2 µM ATO, 0.5-5 ng/ml temsirolimus and the combinations for 24 hours. Whole cell extracts were used in immunoblotting experiments for phospho-S6, S6, phospho-ERK, ERK, and β-actin. Immunoblots shown are representative of experiments performed at least 3 times.

 We next assessed whether inhibition of AKT activity was correlated with the ability of ATO to enhance temsirolimus growth inhibition. Breast cancer cells were treated for 24 hours with temsirolimus, ATO, or the combination, and the expression of total and phosphorylated AKT (serine 473 or threonine 308 phosphorylation) was assessed by immunoblotting. Both phosphorylation sites correspond to activated AKT [12,25]. As expected, temsirolimus increases the levels of phosphorylated AKT, which in MDA-MB-468, MCF-7 and SkBr3 cells, could be inhibited by co-treatment with ATO (Figure 2). In contrast, ATO enhanced temsirolimus-induced phospho-AKT expression in T47D cells. In addition, we analyzed a downstream rapamycin-sensitive mTOR target, phospho-S6 ribosome [26], to confirm that temsirolimus inhibited the mTOR complex and determine the effects of ATO on these downstream targets. Indeed, temsirolimus inhibited S6 phosphorylation in all cell lines in the presence and absence of ATO (Figure 3). Thus, *in vitro*, enhanced growth inhibition of the combination treatment correlated with ATO inhibition of phosphorylated AKT. 

ERK activation may also to lead to rapalog-resistance. We investigated whether ATO decreased these survival signals leading to enhanced growth inhibition in combination with temsirolimus. Interestingly, temsirolimus and ATO as single agents both increased phosphorylation of ERK in MDA-MB-468, MCF-7, SkBr3 and T47D cells, but the combination inhibited ERK phosphorylation (Figure 3). Thus, in cell lines, treatment-induced inhibition of phospho-ERK did not correlate with growth inhibition.

**Figure 4:**
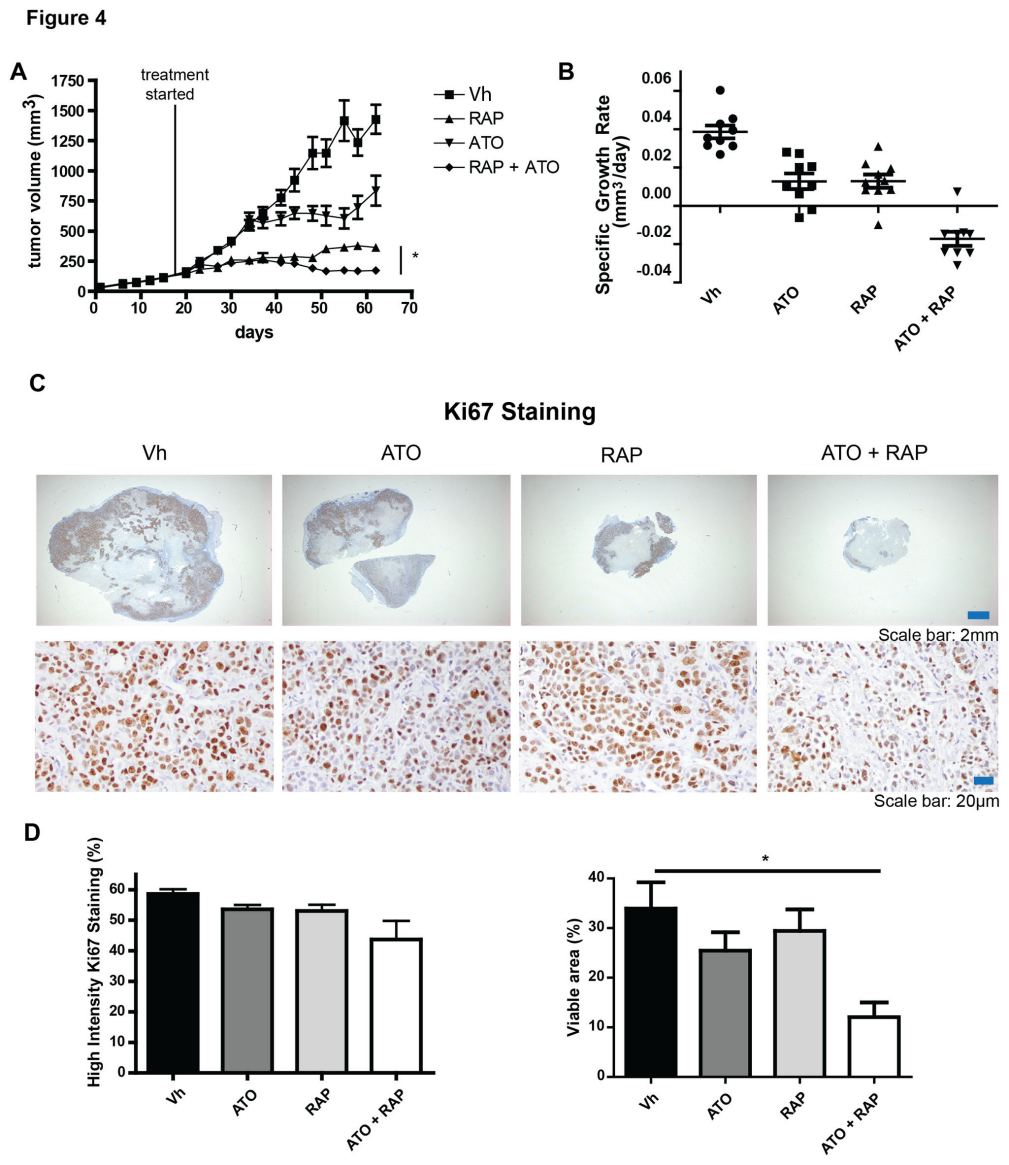
Combination treatment of ATO and rapamycin significantly inhibits tumor growth in an MDA-MB-468 xenograft model. Mice were implanted with MDA-MB-468 cells in the mammary fat pad and once tumors reached 100-200 mm^3^, mice were treated with vehicle (Vh; 20% PEG 400 and 20% TWEEN 80), 7.5mg/kg rapamycin, 7.5 mg/kg ATO, or rapamycin plus ATO every other day. Tumor size was monitored (A) and specific growth rate of the tumor (B) was calculated from days 37-62 and is expressed as change in tumor volume in mm^3^/day. Data are expressed as mean with standard error bars (n = 8-9 mice per group). Tumors were stained with antibodies detecting the proliferative marker Ki67. Representative pictures at low and high magnification of each treatment group are shown (C). Percent cells with high intensity staining and positive Ki67 staining per tumor area were calculated using algorithms provided with the ImageScope software. * = p<0.05 Tumors from individual mice are represented with mean and standard error bars (n = 4-5 mice).

Next, we tested whether ATO could enhance rapamycin-induced tumor killing in a MDA-MB-468 xenograft model, and whether this correlated with markers of response and resistance defined *in vitro*. Tumor cells were implanted in the mammary fat pad and once the tumors reached 100-200 mm^3^, mice were treated with vehicle, 7.5mg/kg rapamycin, 7.5 mg/kg ATO, or rapamycin plus ATO every other day. The timing and dose of rapamycin was previously used in a breast cancer model demonstrating anti-tumor activity [27], and we have previously shown anti-tumor effects with this concentration of ATO [28]. Treatment caused no significant change in animal weight (Figure S1), nor did animals respond adversely to treatment with vehicle alone. After 1 week of treatment, we observed no significant change in hepatic heme oxygenase-1 (HO-1) expression, a marker of ATO-induced oxidative stress [28]. However, at the end of the experiment, ATO treated groups had elevated hepatic HO-1 levels, but this was not further enhanced by the combination with rapamycin (Figure S1). This indicates that the combination treatment is not more hepatotoxic than ATO alone. Next, we assessed tumor regression over 43 days of treatment and found the MDA-MB-468 xenografts responded modestly to ATO, and more significantly to rapamycin, when used as single agents (Figure 4A). However, the ATO plus rapamycin combination significantly improved tumor inhibition as compared to either drug alone. Of note, this enhanced activity was observed in the last 20 days of treatment. Indeed, when we analyzed the specific growth rate for days 41-62 of treatment, vehicle control, ATO, and rapamycin groups all continued to have positive growth rates, while the combination treatment had a negative growth rate (Figure 4B). We then stained tumor section for the proliferative marker Ki67 (Figure 4C). Scanned slides were then analyzed for the percent of nucleated cells that were intensely stained, highly positive cells. There was no significant difference in the percentage of cells with highly intense staining (Figure 4D), however, the percent viable area within the tumor was significantly decreased with the combination treatment (Figure 4D). This suggests that the combination treatment does not reduce proliferation of the residual tumor, but does decrease tumor viability and increase cell death. 

**Figure 5:**
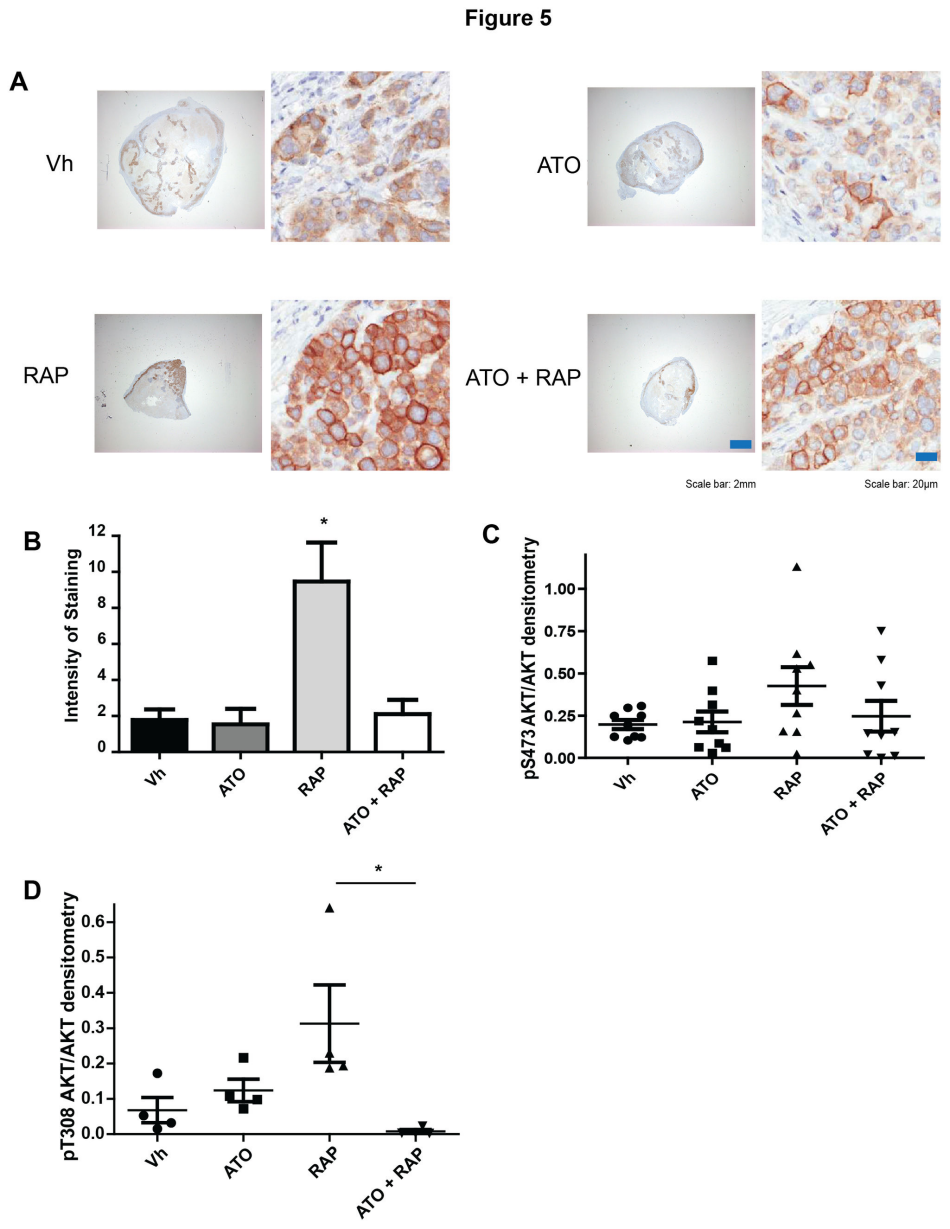
Addition of ATO decreases rapamycin-induced AKT activation *in* *vivo*. Tumor samples were stained with antibody against phospho-S473-AKT. Representative pictures of each treatment are shown (A). Quantification of staining intensity was performed using algorithms provided with the ImageScope software (B). Individual animals are represented with mean and standard error bars (n=4-5 mice). (C-D) Tumors at the end of the experiment were analyzed in immunoblotting experiments with antibodies against phospho-S473-AKT (C), phospho-T308-AKT (D), AKT, and β-actin. Band densitometry was performed and the relative intensity of phospho-AKT/AKT/β-actin was calculated. Individual animals are represented with mean and standard error bars (n= 8-9 mice).

Based on our *in vitro* data, we compared expression of phosphorylated-AKT in tumor samples after treatment with vehicle control, rapamycin, ATO, or the combination of ATO and rapamycin. We assessed phospho-S473 AKT by IHC in tumors after 62 days of treatment. As previously described [6], rapamycin induced the levels of activated AKT, which was then significantly inhibited by concomitant exposure to ATO (Figure 5A & B). We confirmed this result by immunoblot analysis of whole mammary tumor extracts and found that there was a trend toward increased phospho-S473 AKT expression following rapamycin treatment, which was diminished with the combination, although there were no statistically significant differences between the groups (Figure 5C). We postulated that this discrepancy could be due to the increase in necrotic tissue after 62 days of treatment. We did observe that ATO significantly inhibited rapamycin-induced phosphorylation of AKT at T308 in tumor extracts (Figure 5D).

**Figure 6:**
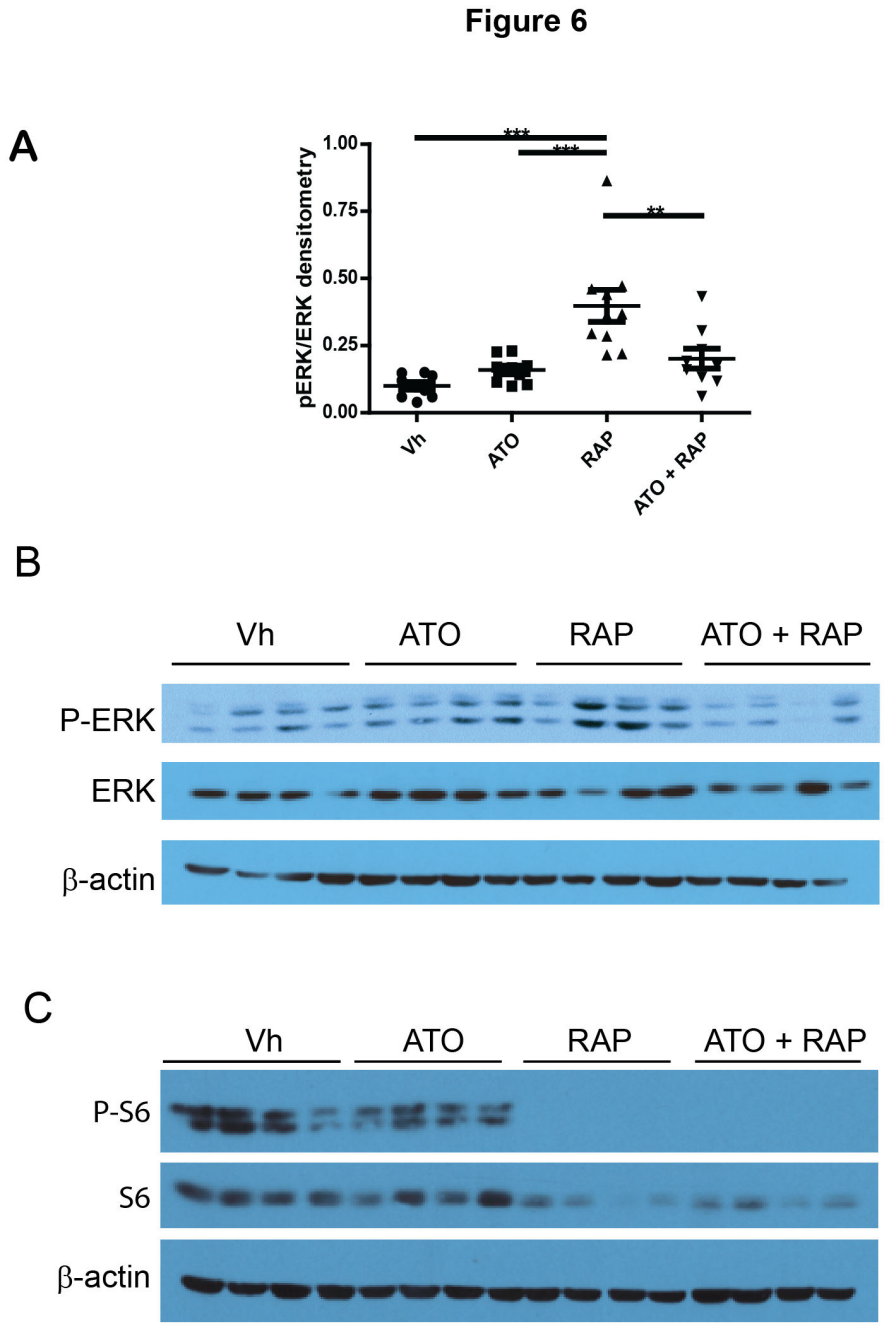
Addition of ATO decreases rapamycin-induced phospho-ERK and phospho-S6. (A-B) Tumors were analyzed in immunoblotting experiments with antibodies against phospho-ERK, ERK, and β-actin. Band densitometry was performed and the relative intensity of phospho-ERK/ ERK/β-actin was calculated. Individual animals (n = 9) are represented with mean and standard error bars. (B) Representative immunoblots of tumors from 4 individual animals per treatment. (C) Tumors were analyzed in immunoblotting experiments with antibodies against phospho-S6, S6, and β-actin. Immunoblots from tumors from 4 individual animals per treatment are shown.

We also assessed phospho-ERK and phospho-S6 in the tumor samples by immunoblotting. In tumors treated for 62 days, phospho-ERK was significantly increased in tumor lysates from rapamycin-treated animals and was significantly decreased in tumors treated with both rapamycin and ATO (Figure 6). The phosphorylation patterns of ERK1 and of ERK2 were similar. These data indicate that ATO plus rapamycin inhibit ERK activation. Furthermore, rapamycin completely inhibited S6 phosphorylation with and without ATO treatment (Figure 6C), indicating that the inhibition of rapamycin-targets is unchanged in the presence of ATO.

## Discussion

The success of rapalogs in the clinical setting has, in part, been hampered by feedback mechanisms, including AKT and ERK activation, which promote growth and survival signals. Combination therapies that inhibit these survival signals are predicted to enhance the efficacy of rapalog treatment. ATO is clinically approved for the treatment of acute promyelocytic leukemia and we have shown previously that ATO functions to inhibit AKT in this context [19]. We now present data in breast cancer cells that ATO inhibits rapalog and rapamycin-induced phosphorylation of AKT and ERK *in vitro* and *in vivo*, respectively. The combination of rapamycin plus ATO significantly impaired tumor growth in a xenograft model, without adding to the hepatotoxicity associated with ATO treatment, beyond the response achieved with rapamycin alone. 

Multiple combination therapies with rapalogs have been, and continue to be, tested in breast cancer [29]. Rapamycin acts additively or synergistically with several chemotherapies including paclitaxel, carboplatin, doxorubicin and gemcitabine to inhibit growth of breast cancer cell lines *in vitro* [30]. *In vivo*, rapamycin enhanced paclitaxel-induced growth inhibitory effects in xenograft models [30]. Everolimus (RAD001) has been tested in combination with paclitaxel in phase I dose and toxicity testing in advanced solid tumors and was found to have an acceptable safety profile [31]. However, addition of everolimus did not enhance the response to paclitaxel in HER2-negative breast cancer patients [32]. No correlative studies with molecular analysis of signaling pathways were published with this study. In contrast, progression-free survival was significantly increased when everolimus was given in combination with an aromatase inhibitor (AI) in hormone-receptor positive breast cancer patients compared to AI therapy alone [33]. These results show that specific populations of breast cancer patients can benefit from combination therapy with rapalogs. 

 The combination of everolimus plus ATO was explored in pre-clinical models of ovarian cancer. Everolimus and ATO acted synergistically to inhibit tumor cell growth *in vitro*, which was associated with concomitant induction of markers of apoptosis and autophagy [34]. In xenograft models, the combination of everolimus and ATO inhibited tumor growth better than either drug alone and markers of enhanced autophagy were observed [34]. Autophagy is a consequence of arsenic exposure in human lymphoblastoid cell lines [35,36]. However, we did not observe markers of increased autophagy (p62, beclin-1, and lamp-2) in lysates of our tumors (data not shown). Intriguingly, we found that the combination of rapalog plus ATO inhibited ERK activation, while each drug alone induced ERK *in vitro*. Alone, ATO can activate ERK [37], but ATO also can inhibit ERK activation induced by other drugs, such as the mitochondrial toxin lonidamine [38] and 2-deoxy-D-glucose [39]. The differential ERK response may be related to whether the outcome of ATO treatment is growth arrest or cell death. In those cells that are growth arrested, ERK may be activated by ATO as a survival mechanism. When apoptosis is induced, this survival mechanism is outcompeted by cell death signals, potentially as a result of JNK signaling [40]. This is certainly the case for the survival signals attributed to p38 MAPK [41]. ERK activation is required for ATO-induced differentiation of APL cells [42], but inhibition of MEK1, and thus ERK, enhances ATO-induced cell death [43,44].

The emergence of resistance mechanisms to single agent therapies confounds the effective treatment of numerous cancers. Indeed, the engagement of feedback mechanisms that blunt the anti-tumor effects of rapalogs is a documented problem with this class of drugs [6,7,11,14]. In this regard, a newer generation of mTOR inhibitors have been developed that target the active site of mTOR and inhibit both mTORC1 and mTORC2. These compounds may overcome the IRS-1/PI3K/AKT feedback loop, but may result in a more profound activation of the ERK pathway [45]. In addition, PI3K inhibitors shut down AKT signaling, but upregulate a compensatory ERK signaling pathway in breast cancer [18]. Our data thus suggest that addition of ATO to the next generation of rapalogs may be beneficial by inhibiting this potential mechanism of resistance.

## Supporting Information

Figure S1 **The combination of ATO plus rapamycin is not more toxic than single agents.** (A) The weight of the tumor-bearing animals treated with vehicle control, 7.5 mg/kg ATO, 7.5 mg/kg rapamycin or the combination were monitored throughout the experimental period. No significant differences were observed. (B) Liver extracts from tumor-bearing animals treated for one week (top) or at the completion of the experiment (bottom) were used to detect heme oxygenase-1 (HO-1), a marker of arsenic-induced oxidative stress. The graphs represent the densitometry of HO-1 expression normalized to β-actin. The combination did not induce more HO-1 than ATO alone.
(TIF)
