# Supplementary figures and images for: Arsenic Trioxide Overcomes Rapamycin-Induced Feedback Activation of AKT and ERK Signaling to Enhance the Anti-Tumor Effects in Breast Cancer
Source: PLoS One. 2013 Dec 31;8(12):e85995. doi: 10.1371/journal.pone.0085995 (PMC3877392; doi:10.1371/journal.pone.0085995)

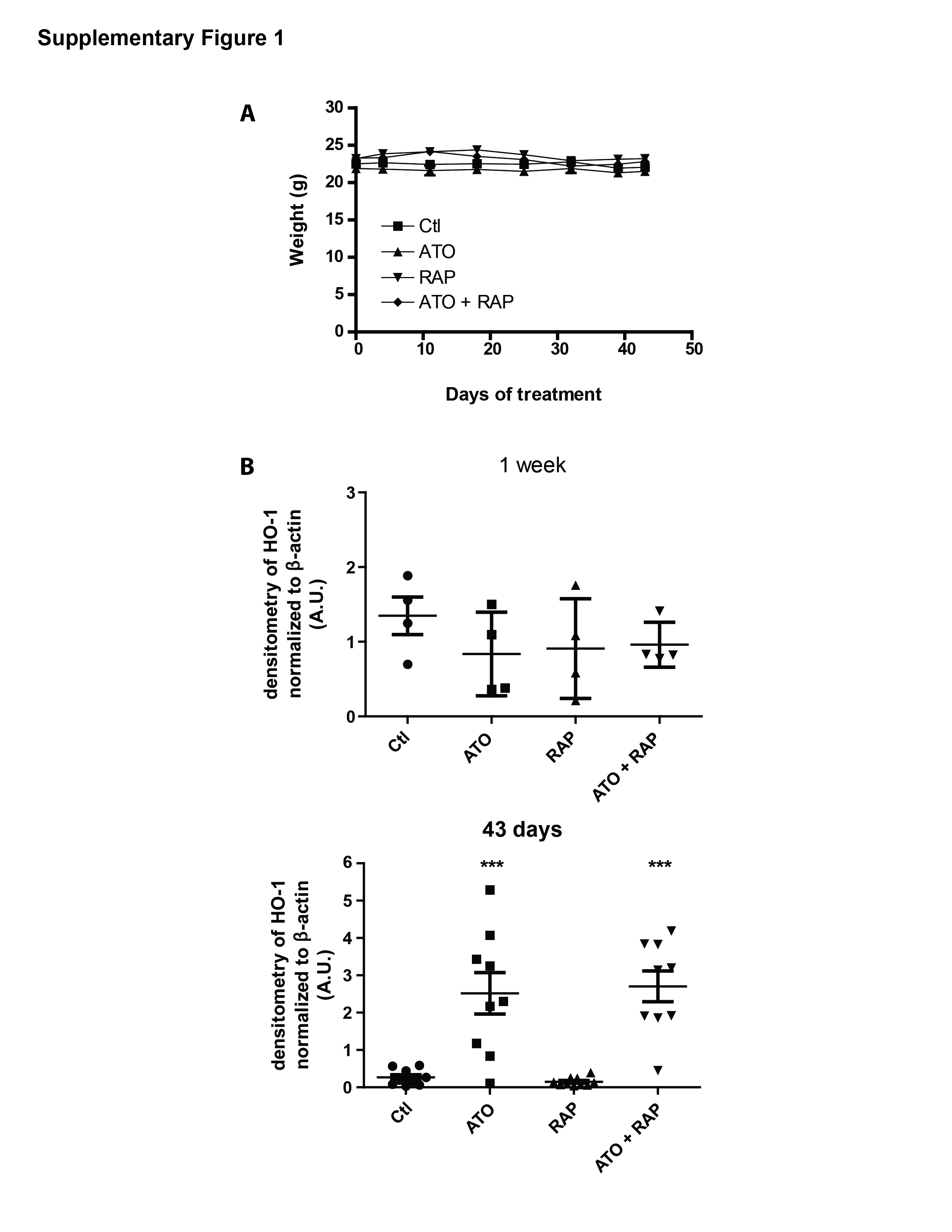

Supplement: Figure S1 — The combination of ATO plus rapamycin is not more toxic than single agents. (A) The weight of the tumor-bearing animals treated with vehicle control, 7.5 mg/kg ATO, 7.5 mg/kg rapamycin or the combination were monitored throughout the experimental period. No significant differences were observed. (B) Liver extracts from tumor-bearing animals treated for one week (top) or at the completion of the experiment (bottom) were used to detect heme oxygenase-1 (HO-1), a marker of arsenic-induced oxidative stress. The graphs represent the densitometry of HO-1 expression normalized to β-actin. The combination did not induce more HO-1 than ATO alone. (TIF) [file pone.0085995.s001.tif]
